# COVID-19 Impacts on Pennsylvania Coordinated Specialty Care for Early Psychosis Participants

**DOI:** 10.1017/dmp.2023.151

**Published:** 2023-09-12

**Authors:** Megan B. E. Jumper (Westfall), Fanghong Dong, Emily M. Becker-Haimes, Lucy Miao, Catherine Conroy, Deepak Sarpal, Courtney Abegunde, Melanie Bennett, Christian G. Kohler, Monica E. Calkins

**Affiliations:** 1 Pennsylvania Early Intervention Center (PEIC)/HeadsUp, Neurodevelopment and Psychosis Section, Department of Psychiatry, Perelman School of Medicine, University of Pennsylvania, Philadelphia, PA, USA; 2 Department of Psychiatry, School of Medicine, University of Pittsburgh, Pittsburgh, PA, USA; 3 Department of Psychiatry, School of Medicine, University of Maryland, Baltimore, MD, USA

**Keywords:** coordinated specialty care, coping, COVID-19 pandemic, early psychosis, psychosocial impacts

## Abstract

**Objectives:**

The coronavirus disease (COVID-19) pandemic produced swift, extensive changes in daily life, including for first-episode psychosis (FEP) clients. This study examined pandemic-related psychosocial impacts to clients while engaged in Coordinated Specialty Care (CSC). We also examined FEP client vaccination rates, as vaccinations can reduce hospitalizations/deaths, and related worries.

**Methods:**

Thirty-one clients (45% female; ages 13-39; 26% black, 61% white) from Pennsylvania (PA) CSC outpatient programs completed an online survey evaluating exposure to COVID-19, associated worries, coping, and safety strategies. Descriptive statistics characterized responses and demographic group differences. Additional program evaluation data informed vaccination rates for PA FEP clients.

**Results:**

Participants reported substantial pandemic-related impacts to daily life. Many clients reported improved safety measures to protect themselves/others from COVID-19. Clients largely denied substantial worries about infection for themselves, reporting greater concern for loved ones. Multiple coping strategies were endorsed, which, with few exceptions, did not differ among demographic groups. FEP clients had a low reported rate of vaccination (28.6%) as of September 2021.

**Conclusions:**

Observed prolonged pandemic effects may alter FEP client progress in CSC. Stakeholders should be prepared to adjust FEP treatment accordingly in the event of a similar disaster. Concentrated vaccination efforts may be necessary for this population.

Pennsylvania (PA) first-episode psychosis (FEP) program evaluation (PE) participants receive care for early psychosis in PA Coordinated Specialty Care (CSC) outpatient programs. CSC programs earned worldwide attention for improved outcomes compared to typical care for early psychosis patients.^1^ PA FEP PE participants demonstrate improved psychiatric symptoms, role and social functioning, decreased hospitalizations, improved self-perceived recovery and quality of life, and service satisfaction by 6- and 12-month follow-ups in PA CSC programs.^2^ In March 2020, the coronavirus disease (COVID-19) pandemic began. Despite continuity of care through a rapid transition to tele-mental health (TMH),^3,4^ there has been escalating international concern about mental health (MH) effects of the prolonged pandemic on the general population,^5^ with special interest in individuals experiencing FEP.^6^ Individuals with FEP can have worsening psychotic experiences during times of stress sensitivity and amplified threat anticipation,^7^ both concerns during the pandemic.

Emerging research reveals adolescents and young adults with psychosis may be exposed to extensive pandemic impacts, including increased infection risks, exacerbation of psychosis and associated symptoms (eg, anxiety, depression, suicidality, substance use), social isolation, occupational dysfunction, and modifications or interruptions in MH treatment access.^8^ This study therefore sought to investigate effects of the pandemic, including perceived psychosocial impacts and coping strategies, both with potential to influence treatment outcomes^8^ specific to the PA early psychosis population (individuals at clinical high risk for developing a psychotic disorder based on recent onset or worsening of sub-threshold psychosis symptoms, or those in their first episode of psychosis). With increased risk of severe COVID-19 for individuals with schizophrenia, including greater hospitalization and mortality rates,^8^ it may be of particular importance for FEP providers and clinicians to help combat COVID-19 vaccine misinformation and hesitancy, providing a secondary aim to report on COVID-19 vaccination rates of FEP participants in comparison to the general population.

## Methods

### Setting

Nine CSC programs for FEP and 2 clinical high risk (CHR) for psychosis programs, which participate in statewide program evaluations (PA-FEP-PE) by HeadsUp at University of Pennsylvania, funded by the Pennsylvania Department of Human Services Office of Mental Health and Substance Abuse Services (PA-OMHSAS), provide services to mental health clients impacted by the pandemic, who served as the primary sample. Each PA-FEP and CHR program follows the CSC model(s)^9^ that best suits agency needs. Programs vary from university hospitals to community-based agencies, with organization-defined inclusion/exclusion criteria. Essential CSC services,^10^ including pharmacotherapy, recovery-oriented cognitive therapy,^11^ case management, Supported Employment and Education (SEE), crisis services, family/caregiver involvement, outreach, psychoeducation, and treatment and discharge planning, are offered by each program for at least 2 years by a multidisciplinary team. CSC interventions’ intensity and frequency for participants were maintained during the pandemic, including TMH appointments, per annual site fidelity review in fall 2020.

### Procedures

In collaboration with University of Maryland, HeadsUp developed online remote self-report surveys for clients at PA CSC programs. Cross-sectional surveys collected respondents’ anonymous demographic information, personal and family COVID-19 exposure, worries, and financial consequences. They were asked to rate (on a 5-point scale ranging from 0 = “not at all” to 4 = “a great deal”) current worries about (1) contracting, (2) dying from, (3) currently having, (4) family member contracting, (5) unknowingly infecting others with, and (6) experiencing significant financial burden following the pandemic.^5^ The survey also captured current details of changes in MH (better/worse/same levels of distress, loneliness, general mental health status) and frequency of utilized coping strategies (work, substance use, emotional support from others, taking action, seeking advice, distracting activities, religion, learning to live with it, prayer/meditation), utilized safety measures (wash hands, cancel/postpone travel, cancel/postpone activities, doctor visits, stockpile food, cleaning and/or medical supplies, avoid high-risk people, avoid public spaces/crowds, prayer, avoid eating out, wear a mask, work/school at home), and severity of impacts to daily life (routines, income/employment, food access, medical care access, MH care access, access to social support systems, and stress levels at home).

Inclusion criteria for surveys were (1) current client of a PA FEP or CHR program, (2) ages 13 and older, (3) access to the Internet, and (4) access to a valid email account. Survey links were distributed remotely to programs through email to program directors/coordinators (N = 20), who were provided with templated email scripts to distribute survey links to program clients (N = 444, as of July 1, 2020). Reliance on program staff to distribute surveys to clients ensured anonymity of respondents and minimized collection of possibly identifiable protected health information (PHI), particularly email addresses. Any individual with access to the link could respond to the survey, and participation was voluntary. Follow-up reminder emails to distribute links to clients who had not yet completed the survey were sent bi-weekly for the duration of the data collection period. Respondents completed remote self-report surveys between June and September 2020. A separate review was conducted in September 2021 on current COVID-19 vaccination status reported by program participants in PA-FEP-PE data.

The protocol was reviewed and approved by the University of Pennsylvania and University of Pittsburgh Institutional Review Boards (IRBs).

Survey data and PA-FEP-PE data were collected and managed using REDCap (Research Electronic Data Capture), hosted at the University of Pennsylvania. Quantitative data were analyzed in R programming, and descriptive statistics summarized responses from surveys. Results were expressed as counts and percentages for categorical variables. Demographic group differences for clients’ levels of pandemic-related impacts, worries, and coping strategies were examined via Fisher’s exact tests.

### Participants

The 31 respondents were participants with FEP or at clinical high risk of psychosis actively enrolled in PA CSC programs. Respondents were 45% female, 55% male; 2% ages 13–17, 74% ages 18–29, 19% ages 30–39; and 26% black, 61% white, 10% other race.

During this stage of the pandemic, stay-at-home orders were lifted across the state of Pennsylvania, but other restrictions remained, including statewide mask mandates, quarantine orders for out-of-state travelers, and largely the continuation of remote work/school. COVID-19 cases and deaths in Pennsylvania varied throughout summer 2020: cases and deaths decreased in earlier summer months but increased again in late July, leading to increased restrictions.

## Results

**Figure 1.**
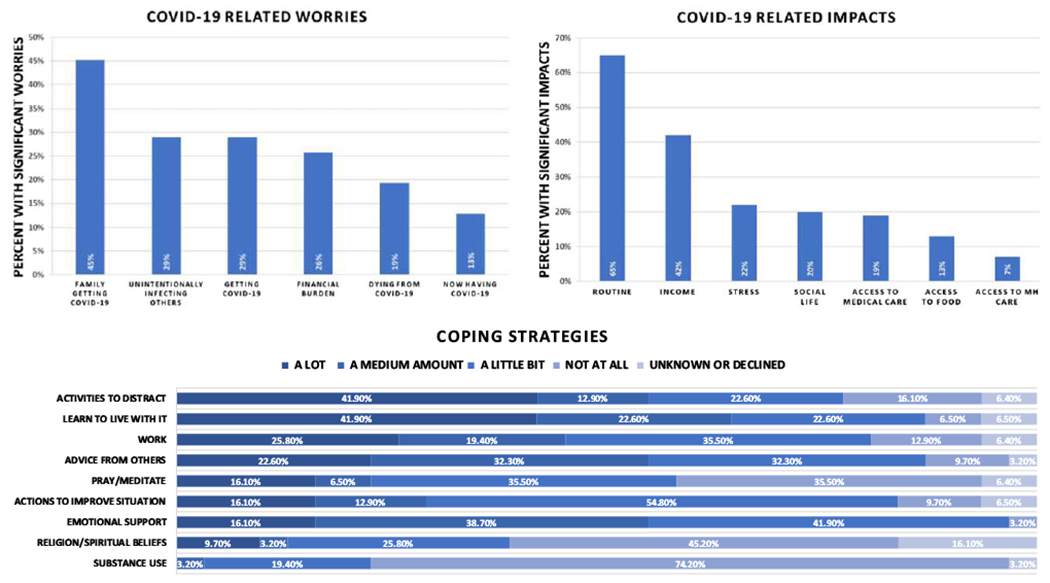
Pandemic-related worries, impacts, and coping strategies reported by PA-FEP-PE program participants. N = 31. Respondents were asked to rate (on a 5-point scale ranging from 0 = “not at all” to 4 = “a great deal”) worries about: (1) contracting, (2) dying from, (3) currently having, (4) family member contracting, (5) unknowingly infecting others with, and (6) experiencing significant financial burden following COVID-19. Significant worries were defined as worries rated at a moderate amount or greater. Participants also reported on pandemic-related impacts and severity of such impacts, where significant impacts were those reported as moderate [3] or greater. Participants also reported coping strategies implemented and frequency of use. Other coping strategies provided by respondents included exercise/walks, cooking, media-use (eg, TV, movies, social, news, video games), reading, and drawing.

Client respondents (N = 31) reported COVID-19 impacts on several aspects of life, especially on daily routine (65%) and household income (42%; Figure 1, top right). A variety of coping strategies were adopted by clients, mainly using distracting activities (41.9%) and learning how to live with the pandemic (41.9%; see Figure 1, bottom). The most common safety measures implemented into routines were washing hands (93.5%), wearing face masks (77.4%), and avoiding public gatherings (54.8%). A small number of clients (10%) reported having been tested for COVID-19. Of clients tested, no respondent reported a positive test result, and most (81%) reported no COVID-19 symptoms. Nonetheless, across groups, many clients were at least moderately worried about family contracting COVID-19 (45%), unknowingly infecting others with COVID-19 (29%), themselves contracting COVID-19 (29%) or dying from COVID-19 (19%), and financial consequences of COVID-19 (26%).

Nearly all demographic groups provided comparable survey responses after examining age, sex at birth, race, and sexual orientation, with a few exceptions. Sex differences were observed for clients who reported at least moderate levels of (1) impacts on access to medical care (female 40%, male 0%, *P* = 0.007), (2) worries about financial strain due to the pandemic (female 46.7%, male 6.3%, *P* = 0.016), and (3) work as a coping strategy (female 66.7%, male 25.0%, *P* = 0.032).

PA-FEP-PE data as of September 2021 revealed 28.6% of participants in PA-FEP programs (N = 161) were vaccinated against COVID-19, with 35.4% unvaccinated and 36.0% preferring not to report vaccination status.

### Limitations

This study has several limitations. Due to the survey’s online format, the sample of respondents was limited to people with Internet access and valid email accounts, and therefore the study does not capture experiences and perceptions of individuals without Internet access, which may markedly differ given socioeconomic reasons for differential Internet coverage. Although responses were anonymous, response bias may have affected the results, particularly about practiced safety measures, COVID-19 exposure, and testing results. Conversely, surveys are subject to nonresponse bias due to their voluntary nature. Ascertaining the level of nonresponse for clients was not possible, given the distribution of surveys was designated to program directors and/or coordinators and they did not collect data on the number of clients to whom survey links were distributed.

Requiring program staff to email the survey to clients strengthened the anonymity of respondents but further limits findings; staff may have shared the survey with only individuals with previously known or easy access and comfort with technology required to complete the survey. The additional burden on staff may also have prevented distribution of the survey to all participants.

## Discussion

Insight into patient worries and perspectives is vital to developing and improving patient-centered mental health care systems.^12^ In addition, expanding availability of vaccinations may reduce adverse outcomes and may influence worries and perspectives around the pandemic. A portion of PA-FEP-PE participants reported at least moderate levels of pandemic-related worries about infection with COVID-19. However, participants indicated greater worry for others than for themselves, including worries about unknowingly infecting others and family members’ health, like the general population.^5^ Participants additionally reported at least moderate levels of pandemic-related impacts, particularly in areas of daily routine, income, and stress. Previous studies indicated that pre-existing psychiatric disorders may be a risk factor for developing or exacerbating additional symptoms, such as anxiety and posttraumatic stress disorder,^13^ and have shown that stress sensitivity and enhanced threat anticipation are associated with more intense psychotic experiences in FEP patients.^7^ Prolonged pandemic-related worries and impact in daily life observed in this study for FEP clients conceivably may amplify psychotic symptoms, interfere with ability to obtain medical or MH care, and/or achieve improvements typically observed during treatment in PA CSC programs. Given clients’ pandemic-related worries and the pervasive impact of the COVID-19 pandemic on daily life, it may be important to monitor for and provide additional support to this vulnerable population in the case of disaster-related mental health problems.

Participants’ most frequently reported coping strategies to deal with pandemic-related stress were activities to distract, learning to live with it, and work—all strategies that relatively lack emotional or social support. Further investigation is needed to determine whether specific pandemic coping strategies correlate to changes to previously witnessed clinical and functional improvements in PA CSC programs.^2^ As the pandemic continues, providers for the FEP population may need to address the lack of psychosocial support used by participants to cope with pandemic-related stressors.

It is of additional importance to acknowledge potential sex-related differences on pandemic-related impacts, stressors, and coping mechanisms. In this sample, female respondents more frequently reported worries about financial strain and medical care access, and utilization of work as a coping strategy, compared to males. Female FEP clients may require additional consideration when approaching CSC treatment during a pandemic or wide-spread disaster.

Despite reported worries about familial COVID-19 cases and unknowingly infecting others, PA FEP participants were vaccinated at a lower rate (28.6%) than the general PA population (55.5% fully vaccinated).^13^ Additionally, high rates of refusal to disclose vaccination status may imply discomfort or anxiety with discussions around vaccination. Given that rates of hospitalization and severe COVID-19-associated illness are reduced by vaccination, which may additionally reduce pandemic-related anxieties, it will be important for FEP clinicians to address vaccine misinformation and hesitancy in the FEP population, including in PA CSC programs.

## Conclusion

Pandemic-related worries, coping and safety strategies, as well as vaccination rates may impact the future of FEP care. During times of disaster, it will be imperative for clinicians and providers to address magnified stress and impacts on this vulnerable population in mental health treatment. Policy-makers need to allow flexibility in CSC program interventions to ensure FEP clients continue to make treatment progress during times of large-scale distress, such as a pandemic. Additionally, clinicians and policy-makers alike may need to concentrate vaccine education and administration efforts in this population, given the observed low vaccination rate. Further data collection and analyses on COVID-19 impacts for the FEP population in PA will allow expansion of current findings. This information will be useful to stakeholders at all levels, including clients, families, clinicians, behavioral health organizations, and state-level policy and funding bodies.

## Abbreviations:

CHR: clinical high risk
CSC: Coordinated Specialty Care
FEP: first-episode psychosis
HHS: US Department of Health and Human Services
IRB: Institutional Review Board
MH: mental health
PA: Pennsylvania
PA-OMHSAS: Pennsylvania Office of Mental Health and Substance Abuse Services
PE: program evaluation
PHI: protected health information
SAMHSA: Substance Abuse and Mental Health Services Administration
SEE: Supported Employment and Education
TMH: tele-mental health
